# An integrated automated deep learning framework for annotating tumor-infiltrating lymphocytes in lung adenocarcinoma pathology

**DOI:** 10.3389/fbinf.2026.1764743

**Published:** 2026-03-16

**Authors:** Xia Li, Kang-Lai Wei, Zhao-Quan Huang, Zi-Yan Huang

**Affiliations:** 1 Department of Pathology, The First Affiliated Hospital of Guangxi Medical University, Nanning, Guangxi, China; 2 Department of Pathology, The Second Affiliated Hospital of Guangxi Medical University, Nanning, Guangxi, China; 3 Health Management Department, The First Affiliated Hospital of Guangxi Medical University, Nanning, Guangxi, China

**Keywords:** automated annotation, deep learning, lung adenocarcinoma, pathology, tumor-infiltrating lymphocytes

## Abstract

**Objective:**

Quantitative analysis of tumor-infiltrating lymphocytes (TILs) is crucial in computational pathology studies of lung adenocarcinoma. However, acquiring large-scale, fully annotated datasets remains a major obstacle for the supervised learning approaches that currently dominate high-precision modeling. To address this data bottleneck, we developed a fully automated pipeline for the precise annotation of tissue contours, tumor parenchyma, and lymphocytes in whole-slide images (WSIs).

**Methods:**

This study utilized WSI data from The Cancer Genome Atlas (TCGA) cohort, with comprehensive manual annotations performed by two pathologists using QuPath software, with all annotations subsequently reviewed by a third senior pathologist. The resulting training dataset comprised over 20,000 annotated units. These annotated data were used to train three core modules consisting of an OpenCV-based image processing pipeline for tissue contour detection, a lightweight U^2^-NetP model for tumor parenchyma segmentation, and a YOLOv7 object detection framework for TILs identification within stromal regions. The pipeline was rigorously validated on both an independent internal cohort and an external hospital cohort, and its outputs were benchmarked against semi-quantitative assessments from expert pathologists.

**Results:**

The pipeline demonstrated robust and generalizable performance. For tissue contour detection, the OpenCV-based pipeline achieved a Dice coefficient of 90.90% on the test set. For the core learning-based tasks, the tumor parenchyma segmentation model achieved a Dice coefficient of 87.17% on the internal test set and maintained consistent accuracy on the external cohort, with Dice coefficients ranging from 0.8509 to 0.9178. In the particularly challenging task of lymphocyte detection, the YOLOv7-based model attained an F1-score of 78.84% and mAP@0.5 of 81.16% on the test set, with performance sustained on external data. Critically, the automated TILs quantifications showed excellent agreement with independent pathologist assessments (ICC >0.96). The implementation of optimized lightweight architectures enables the pipeline to serve as an accessible solution for large-scale WSIs analysis in computational pathology.

**Conclusion:**

This study has successfully developed a fully automated annotation pipeline for lung adenocarcinoma WSIs. By generating high-quality annotations of stromal TILs, this pipeline establishes a reliable data foundation for subsequent computational pathology research and facilitates the advancement of artificial intelligence applications in pathology.

## Introduction

Tumor-infiltrating lymphocytes (TILs), as key effector cells in the tumor immune microenvironment, exhibit spatial distribution and density patterns in lung adenocarcinoma (LUAD) that hold significant predictive value for disease progression, patient prognosis, and response to immunotherapy (Zhang et al., 2023; Nguyen et al., 2022). Computational pathology-based quantitative analysis of TILs is emerging as a promising approach to better understand their role in tumor immunity and explore potential clinical applications (Kraja et al., 2025). In this field, supervised learning using extensively annotated whole-slide images (WSIs) remains the primary method for developing high-precision computational models (Ian Goodfellow and Courville, 2016). However, LUAD often exhibits widespread and complex lymphocytic infiltration, resulting from chronic inflammatory stimulation due to tumor invasion and the anatomical exposure of lung tissue to the external environment (Desharnais et al., 2025; Mughal et al., 2025). Under such persistent stimulation, lymphocytes frequently show substantial heterogeneity in size and morphology, creating significant annotation challenges, including heavy workloads and inconsistencies among different annotators.

While emerging approaches such as weakly supervised learning and cross-institutional standardized annotation protocols (Jiang et al., 2022) can substantially reduce the annotation burden, enabling semi-supervised or even unsupervised model training, they still face technical constraints, particularly limited model generalizability. Consequently, these methods have not yet fundamentally addressed issues of annotation consistency and reliability. This situation underscores the pressing need to develop efficient and reliable automated annotation models specifically designed for TILs.

The integration of artificial intelligence, particularly recent advances in deep learning for medical image analysis, has created new opportunities for automated TILs annotation. These automated tools not only improve throughput in histopathological examinations and reduce inter-observer variation (Baxi et al., 2022) but also significantly alleviate pathologists' repetitive workloads. Several relevant studies have contributed to this field. For instance, Karina Silina et al. (Silina and Ciompi, 2025) developed HookNet-TLS, a deep learning algorithm for automatically identifying and quantifying lymphoid aggregates—including tertiary lymphoid structures with germinal centers—in cancer tissue sections. More recently, Li et al. (Li et al., 2025) introduced a deep learning model that combines self-supervised learning with domain adaptation to enable automated classification and annotation of multiple cellular components in H&E-stained histopathological images. Nevertheless, these models still depend on high-performance GPUs and involve time-consuming processes with substantial computational demands, which somewhat restricts their widespread adoption and practical implementation.

Building on the powerful feature extraction capabilities of deep learning in pathological image analysis, this study developed an integrated multi-model automated annotation pipeline. By effectively combining multiple specialized deep learning networks, our pipeline automatically identifies TILs in LUAD-WSIs while simultaneously performing precise segmentation of tissue contours and accurate delineation of tumor parenchymal regions. This integrated design ensures that the generated annotation data accurately capture lymphocyte spatial localization, making it particularly suitable for quantitative and spatial distribution analysis of TILs within the tumor stroma. Additionally, the pipeline employs a lightweight architecture that maintains high annotation accuracy while significantly improving analytical efficiency and reducing computational resource requirements. This approach effectively addresses the critical challenge posed by the scarcity of high-quality annotated datasets.

## Methodology

### Data collection

A total of 503 LUAD cases were retrieved from The Cancer Genome Atlas (TCGA) database, of which 314 had available WSIs. All WSIs were derived from routinely stained pathological sections; frozen sections were excluded to maintain staining consistency. Two experienced pathologists evaluated all WSIs and applied the following exclusion criteria:

1. abnormal staining (e.g., over-stained or faded);
2. impaired tissue integrity (e.g., folds, overlaps, or tears);
3. image quality issues (e.g., pen marks, blurring, or insufficient magnification).

Following this quality assessment, five eligible WSIs were randomly selected for full-slide manual annotation to constitute the core training set. Additionally, to construct an independent internal validation cohort, ten further eligible TCGA-LUAD WSIs were randomly selected, ensuring no patient overlap with the five training WSIs. To evaluate cross-institutional generalizability, an external validation cohort comprising five LUAD WSIs was obtained from the Second Affiliated Hospital of Guangxi Medical University, representing a separate source with differing staining and scanning protocols.

### Manual annotations

Annotations were systematically performed using QuPath software (v0.5.1). Two pathologists, each with over 3 years of experience in thoracic oncology pathology, independently performed the following tasks:

1. Delineation of the entire tissue boundary;
2. Precise annotation of all tumor regions (including primary and metastatic foci) using the polygon tool;
3. Point-based labeling of individual lymphocytes within the tumor stroma, particularly at the tumor-stroma interface;
4. Delineation and classification of necrotic areas (distinguishing between central necrosis and scattered necrotic foci) and hemorrhagic areas (differentiating fresh from old hemorrhage).

All annotations were reviewed by a third senior pathologist. Any discrepancies were resolved through discussion until a consensus was reached. The final annotation data were exported and saved in JSON format.

### Data preprocessing

A systematic data preprocessing pipeline was implemented to enhance image quality and improve model generalizability. First, Gaussian and median filtering were applied to reduce noise, while interpolation and neighboring pixel filling were employed to reconstruct missing regions. Subsequently, an extensive data augmentation strategy was adopted, comprising random horizontal/vertical flipping, 90°rotation, random cropping (85%–100% of original area), and perturbations in HSV color space (hue: ±10%, saturation: ±15%, value: ±10%). This approach improved model robustness to variations in imaging conditions. Finally, the dataset was partitioned at the whole-slide level into training (70%), validation (15%), and test (15%) sets to prevent data leakage and ensure independent evaluation.

### Implementation and optimization of the tissue contour segmentation pipeline

To efficiently process high-resolution WSIs, a multi-scale preprocessing strategy was implemented for tissue segmentation. First, WSIs were uniformly downsampled to a 2.5×objective resolution to create low-resolution thumbnails for rapid processing. The thumbnails were converted to grayscale, and the Roberts edge detector was applied to highlight tissue-background boundaries. The Otsu adaptive thresholding algorithm was then used to binarize the edge map, generating initial candidate tissue regions.

**FIGURE 1:**
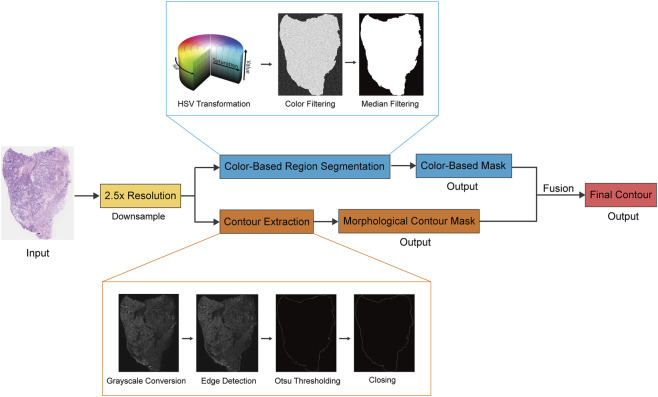
Flowchart of the tissue contour segmentation pipeline based on OpenCV.

To enhance segmentation robustness, morphological closing with a 3 × 3 circular structuring element was performed three times to fill holes and repair boundary discontinuities caused by uneven staining or noise. To further suppress non-tissue artifacts (e.g., glass background, folds, and bubbles), the original image was converted to HSV color space, and H&E-stained pink/purple tissue regions were extracted by thresholding (H: 18–180, S: 9–255, V: 0-255). A median filter (radius: 4 pixels) was applied to the color-filtered result to eliminate small residual noise. Finally, the candidate mask from edge detection and the color space filtering result were merged via a logical AND operation to produce a continuous, complete tissue region mask (Figure 1).

### Tumor parenchyma segmentation model training

**FIGURE 2:**
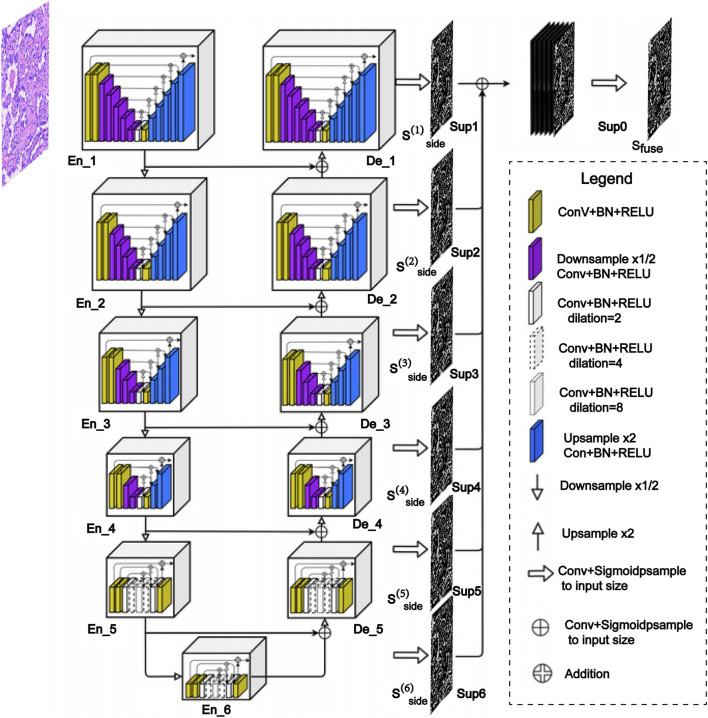
Schematic diagram of the U^2^-NetP architecture for tumor parenchyma segmentation.

Accurate segmentation of tumor parenchyma is challenging due to its intricate interweaving with stromal regions, poorly defined boundaries, and high tissue heterogeneity. To address this, we employed a lightweight U^2^-NetP model (Figure 2). Based on a nested U-shaped architecture, the model utilizes residual U-blocks (RSUs) for hierarchical multi-scale feature extraction. Deep RSU modules capture fine-grained local features such as nuclear morphology and glandular lumina, whereas shallow RSUs integrate broader structural information including gland distribution patterns and stromal fiber orientation. By fusing dilated and standard convolutions, the RSUs enlarge the receptive field while maintaining spatial resolution, effectively capturing features from cellular details to tissue-level architecture. This lightweight design preserves the model’s ability to represent complex tissue structures while reducing computational overhead, making it well-suited for efficient WSIs analysis.

To align with task requirements, input images were rescaled to a resolution equivalent to 5×objective magnification. This scale optimally balances computational efficiency with the preservation of structural detail, providing the model with sufficient context for tissue layout and architecture. A deliberately designed cross-level feature fusion mechanism enables the model to effectively integrate local patterns (e.g., acinar structures and cell density) with global morphological information (e.g., collagen fiber orientation and necrotic area distribution). This integration significantly improves segmentation performance in areas with ambiguous boundaries and high tissue heterogeneity.

During decoding, a lightweight attention-guided feature fusion strategy was incorporated to enhance feature responses in salient regions and improve boundary precision. For model training, 640 × 640-pixel image patches were used as input, with a batch size of 16 to fit GPU memory constraints and maintain gradient stability. We used the AdamW optimizer with hyperparameters β_1_ = 0.9, β_2_ = 0.999, andε = 1 × 10^−8^. The initial learning rate was set to 1 × 10^-4^and dynamically adjusted via a cosine annealing scheduler (T_max = 50). The overall loss function combines weighted binary cross-entropy loss and Dice loss to simultaneously address class imbalance and ensure accurate boundary segmentation. The total loss is defined as follows:

$$L_{total}=\alpha \;\cdot \;L_{WBCE}+\beta \;\cdot \;L_{Dice}$$

where α and β are weighting coefficients that balance the two loss terms.

The weighted binary cross-entropy loss is defined as:

$$L_{WBCE}=-\frac{1}{N}\sum_{i}\left[w\;\cdot \;y_{i}\;\cdot \;\log\left({ŷ}_{i}\right)+\left(1-y_{i}\right)\;\cdot \;\log\left(1-{ŷ}_{i}\right)\;\right]$$

Here, yᵢ and ŷᵢ denote the ground truth label and predicted probability of the *i*th pixel, respectively, w is the weight for positive (foreground) samples, and N is the total number of pixels.

The Dice loss, based on the F1-score, measures the overlap between predictions and ground truth:

$$L_{Dice}=1-\frac{2\sum_{i=1}^{N}y_{i}{ŷ}_{i}+\epsilon }{\sum_{i=1}^{N}y_{i}+\sum_{i=1}^{N}{ŷ}_{i}+\epsilon }$$

where ∈ is a smoothing term (set to 1 × 10^−6^) included to prevent division by zero.

The total number of training epochs was set to 300, and an early stopping strategy (patience = 20) was applied to prevent overfitting. Metrics including F1-score, Precision, and Recall were monitored on the validation set throughout the training process.

### Lymphocyte detection model training

The localization and identification of lymphocytes present three major challenges: (1) morphological similarity to tumor cell nuclei, with an average diameter difference of < 2 μm, complicating their distinction through traditional image processing; (2) severe cell overlap, especially in high-density regions (>1500 cells/mm^2^); and (3) the frequent presence of tertiary lymphoid structures formed by lymphocyte aggregation.

**FIGURE 3:**
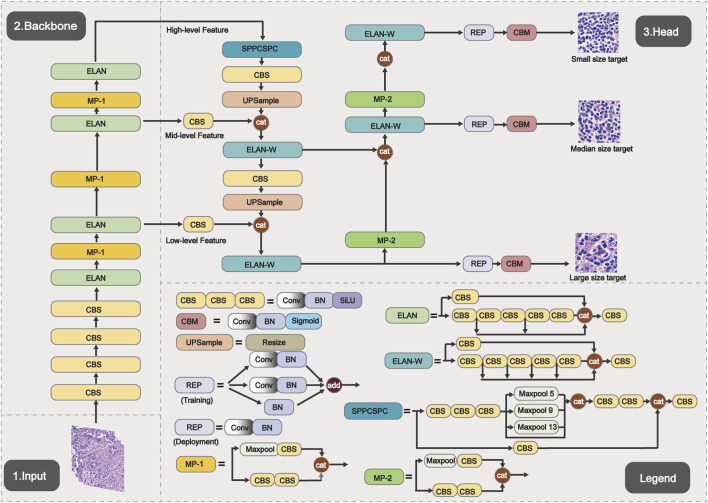
Schematic diagram of the YOLOv7 model architecture.

To address these challenges, the YOLOv7 object detection architecture was adopted (Figure 3). The model integrates an E-ELAN backbone with an FPN + PAN neck, enabling multi-scale feature fusion that captures both local cellular textures and global distribution patterns. A decoupled detection head is used to separately optimize classification and bounding box regression, while an anchor-based mechanism combined with non-maximum suppression effectively reduces duplicate and missed detections in high-density cell regions. To enhance sensitivity to small targets, multi-scale prediction layers (P3-P5) were incorporated, substantially improving lymphocyte detection performance.

During training, input images were resized to 640 × 640 pixels with a batch size of 8. Optimization was performed using stochastic gradient descent (SGD) with an initial learning rate of 1 × 10^−2^, weight decay of 5 × 10^−4^, and a cosine annealing learning rate scheduler for improved stability. The loss function consisted of binary cross-entropy (BCE) loss for the classification branch, Complete IoU (CIoU) loss for bounding box regression, and BCE loss for the object confidence branch. The CIoU loss, which incorporates overlap area, center-point distance, and aspect ratio, is defined as follows:

$$L_{CIoU}=1-IoU+\frac{p^{2}\left(b,b^{gt}\right)}{c^{2}}+\alpha v$$

Here, b and b_gt denote the center points of the predicted and ground-truth bounding boxes, respectively; ρis the Euclidean distance between them; c represents the diagonal length of their minimal enclosing box; v quantifies aspect ratio consistency; andαdenotes a weighting function.

The total number of training epochs was set to 250, and metrics including Precision, Recall, and F1-score were monitored on the validation set throughout the training process.

### Experimental setup and training monitoring

All experiments were performed on a computational platform equipped with an NVIDIA Tesla A100 40 GB GPU and an Intel Xeon Gold 6226R CPU, using Python 3.6.9, PyTorch 1.8.0, CUDA 11.1, and cuDNN 8.0.5. Training was monitored in real time via TensorBoard to track loss curves and performance metrics. Model weights and training logs were saved every 20 epochs. The final model was selected according to the highest Dice coefficient and F1-score on the validation set and used for subsequent inference.

### Model evaluation metrics

To systematically evaluate model performance, task-specific metrics were employed according to the requirements of each task. The Dice similarity coefficient served as the primary metric for tissue contour detection and tumor parenchyma segmentation, quantifying spatial overlap at the pixel level to assess boundary consistency. For lymphocyte detection, the F1-score, which balances precision and recall, was used as the main criterion to evaluate classification performance at the cellular level. Here, precision (the proportion of correct positive predictions) reflects the model’s reliability in avoiding false alarms, whereas recall (the proportion of actual positives correctly identified) indicates its completeness in detecting targets. In addition, for the YOLOv7 model, the mean Average Precision at an Intersection-over-Union threshold of 0.5 (mAP@0.5) was adopted to further assess detection accuracy under this commonly used localization criterion. Additional metrics, including accuracy, precision, and recall, were also reported to enable comprehensive performance analysis. All metrics were derived from the confusion matrix, which comprises true positives, true negatives, false positives, and false negatives; detailed definitions are provided in the Supplementary Material.

### Inter-rater agreement assessment protocol

To validate the clinical relevance of the automated TILs quantification, we compared the pipeline’s outputs against semi-quantitative assessments from two independent pathologists using the independent internal validation cohort (ten TCGA-LUAD WSIs, see Data Collection). For each WSI, TILs counts were computed based on automated detection results and independently by both pathologists through manual counting across five high-power fields (HPFs, 400×). Continuous count values were used to calculate the intraclass correlation coefficient (ICC) under a two-way random-effects model for absolute agreement. Subsequently, count values were categorized into three grades: low, moderate, and high. The threshold values for these categories (<100, 100–300, and >300 cells/HPF, respectively) were determined retrospectively based on the distribution of manual TILs counts provided by the two expert pathologists in this study, ensuring optimal alignment with their independent grading consensus. Agreement in categorical grading was assessed using Cohen’s kappa for pairwise comparisons, and overall multi-rater agreement was evaluated with Fleiss’ kappa. Bland-Altman plots were generated to visualize systematic biases between raters.

### External validation cohort and evaluation protocol

To rigorously assess the cross-institutional generalizability of our pipeline, we performed a comprehensive evaluation using the external validation cohort (10 WSIs from the Second Affiliated Hospital of Guangxi Medical University, covering five major histological subtypes: solid, papillary, complex acinar, acinar, and lepidic patterns; see Data Collection).

Given that the tissue contour segmentation is implemented by a deterministic, non-learning-based OpenCV pipeline (as detailed in the Methodology), which demonstrated near-perfect performance on the internal test set, external validation was focused on the core learning-based modules: tumor parenchyma segmentation and lymphocyte detection.

For tumor parenchyma segmentation, the annotated tumor-stroma regions across all WSIs were systematically divided into 319 non-overlapping patches (500 × 500 pixels each). For lymphocyte detection, within each patch, the TILs count generated by our YOLOv7-based detector was compared against the manual count derived from point-based annotations performed by an experienced thoracic pathologist. Agreement was quantified using the ICC under a two-way random-effects model for absolute agreement, with 95% confidence intervals calculated.

### Research flowchart

**FIGURE 4:**
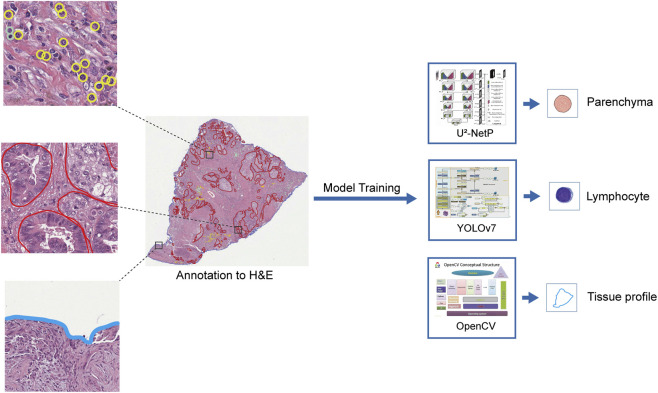
Overall study design and workflow for automated cell annotation.

The overall study design and workflow for automated cell annotation are illustrated in Figure 4.

## Results

### Construction of high-quality annotated dataset

To establish a reliable dataset for cell morphology learning, full-slide manual annotation was performed on selected LUAD-WSIs. Tissue contours, tumor parenchyma, invasive foci, and lymphocyte distributions were precisely delineated using specialized annotation software. The resulting dataset, constructed from WSIs in the TCGA repository, contains over 20,000 annotated units for deep learning model training. Here, an “annotated unit” refers to a distinct region or object that was manually outlined or marked with a point label within regions of interest (ROIs) using the annotation software, each assigned a specific class label. Specifically, these units include 5 tissue contours, 16,140 TILs and 3,840 tumor parenchymal regions categorized by histological subtype: papillary (n = 1,752), acinar (n = 651), solid (n = 752), and lepidic (n = 740) patterns.

### Overall performance of the integrated annotation workflow

Our integrated pipeline comprised three specialized modules: a tissue contour segmentation pipeline, a tumor parenchyma segmentation model, and a lymphocyte detection model. The overall performance of these modules on the test set is summarized in Table 1. Following this summary, we present detailed analyses and visualizations for each module.

**TABLE 1:** Overall performance evaluation of the three core modules in the annotation workflow.

Module	Task	Dice coefficient(%)	F1-score (%)	Precision (%)	Recall (%)	mAP@0.5 (%)
OpenCV-based pipeline	Tissue contour segmentation	90.90	\	92.64	90.45	\
U^2^-NetP	Tumor parenchyma segmentation	87.17	\	87.30	88.93	\
YOLOv7	Lymphocyte detection	\	78.84	77.89	79.81	81.16

### Tissue contour segmentation and visualization pipeline

**FIGURE 5:**
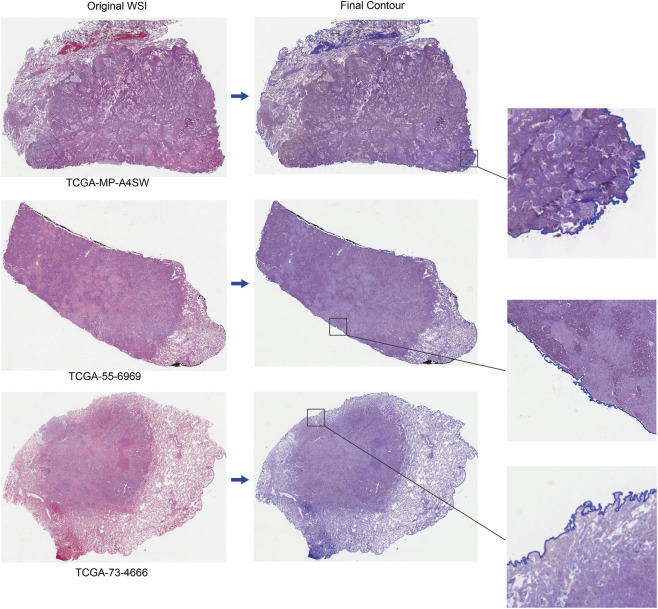
Visualization of the tissue contour generation pipeline and results. The left column shows the original WSIs, and the right column displays the final tissue contour annotations. Each row represents an independent sample, with an inset in the lower-right corner providing a zoomed-in view of a randomly selected corresponding region.

Tissue contour detection established the spatial foundation for all subsequent analyses. We employed a standard image processing pipeline based on OpenCV, which consists of optimized algorithms for edge detection and morphological operations. This pipeline demonstrated strong agreement with pathologist annotations, accurately identifying complex tissue structures such as fibrous connective tissue margins and low-contrast regions. Furthermore, the pipeline maintained robust segmentation performance even in challenging cases including scattered small tissue fragments. Figure 5 shows the annotation results of tissue contours generated by the model across multiple images. Performance evaluation of the pipeline showed that it achieved a precision of 92.64%, recall of 90.45%, and Dice coefficient of 90.90% on the test set.

### Systematic evaluation and architecture selection for tumor parenchyma region segmentation

Within the precisely delineated tissue regions, the tumor parenchyma segmentation model effectively distinguished tumor from stromal areas. To identify the optimal architecture for this task, a systematic comparison was conducted among several representative models: U^2^-NetP, U-Net, DeepLabV3, and PSPNet, which collectively represent mainstream technical routes in current image segmentation research. U-Net serves as a benchmark in medical image segmentation due to its classic encoder-decoder structure with skip connections. DeepLabV3 captures multi-scale contextual information through atrous convolutions and atrous spatial pyramid pooling, providing adaptability to tumor regions of varying sizes. PSPNet utilizes pyramid pooling modules to aggregate global context, enhancing the recognition of structural patterns across different LUAD subtypes.

**FIGURE 6:**
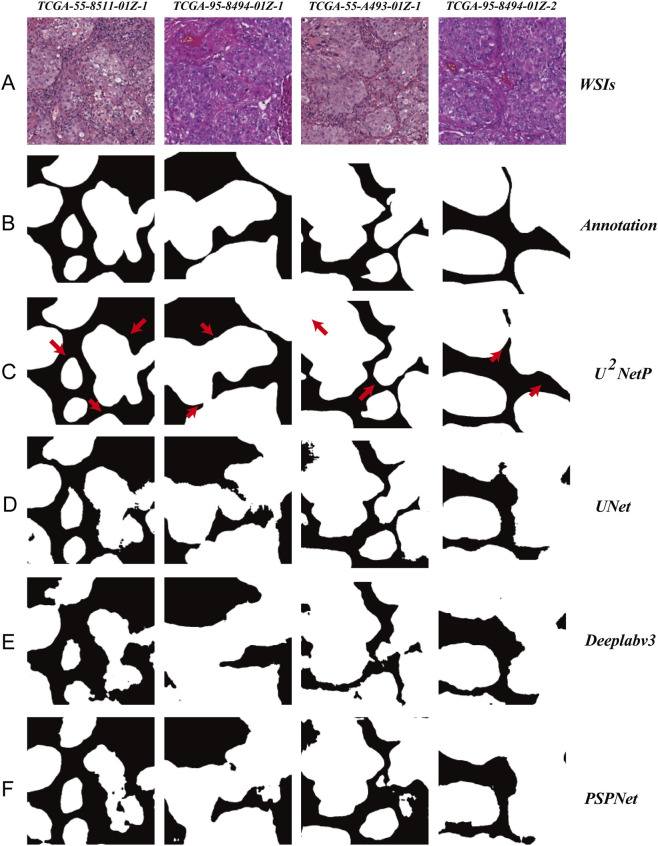
Comparative performance of deep learning architectures in LUAD parenchyma segmentation. **(A)** Representative H&E-stained tissue region. **(B)** Manual annotation (ground truth mask). **(C–F)** Prediction masks from different models: **(C)** U^2^-NetP, **(D)** U-Net, **(E)** DeepLabV3, **(F)** PSPNet. Black and white indicate tumor parenchyma and stroma, respectively. Red arrows highlight regions where U^2^-NetP achieves superior recognition.

Comparative results demonstrate that U^2^-NetP achieves an optimal balance between segmentation accuracy and computational efficiency. Visual analysis further reveals its superior performance in maintaining segmentation consistency along ambiguous tumor-stroma boundaries, with accurate identification of complex growth patterns such as lepidic growth. In contrast, U-Net exhibited over-segmentation in small tumor island regions, DeepLabV3 demonstrated limited sensitivity to low-contrast boundaries, and PSPNet produced fragmented segmentations in morphologically heterogeneous areas. On the TCGA-LUAD test set, U^2^-NetP attained a Dice coefficient of 87.17%, significantly outperforming U-Net (84.32%), DeepLabV3 (85.61%), and PSPNet (83.95%). Notably, while achieving the highest segmentation accuracy, U^2^-NetP maintains only 1.13M parameters with a model size of 4.60 MB, substantially lower than all comparative models (Table 2; Figure 6). This lightweight design enables accelerated inference speed, enhancing its practicality for clinical deployment.

**TABLE 2:** Performance comparison of different architectures in tumor parenchyma segmentation task.

Model	Params (M)	FLOPs (G)	Dice (%)	Inference speed (FPS)	Model size (MB)
UNet	25.47	13.35	84.42	92.5	94.9
Deeplabv3	5.81	3.05	81.70	67.9	22.4
PSPNet	2.37	1.24	80.51	63.5	9.30
U^2−^NetP	1.13	0.59	87.17	36.7	4.60

Params = Number of trainable parameters (unit: M). FLOPs, Floating point operations (unit: G, 10^9 operations). Inference speed = Frames processed per second (tested on NVIDIA, Tesla A100 GPU). All models were trained and evaluated under identical experimental conditions using the same TCGA-LUAD, dataset.

### Segmentation performance and multi-scenario evaluation of tumor parenchymal regions

Based on the comprehensive experimental evaluation, U^2^-NetP was selected as the core architecture for tumor parenchyma segmentation. The implemented model effectively addresses key challenges including ambiguous tumor-stroma boundaries and regional heterogeneity through its multi-scale feature fusion mechanism, which enables precise analysis spanning from cellular-level textures to tissue-level structures. On the test set, the model achieved a precision of 87.30%, recall of 88.93%, and a Dice coefficient of 88.11%, confirming the rationale for this architectural selection.

**FIGURE 7:**
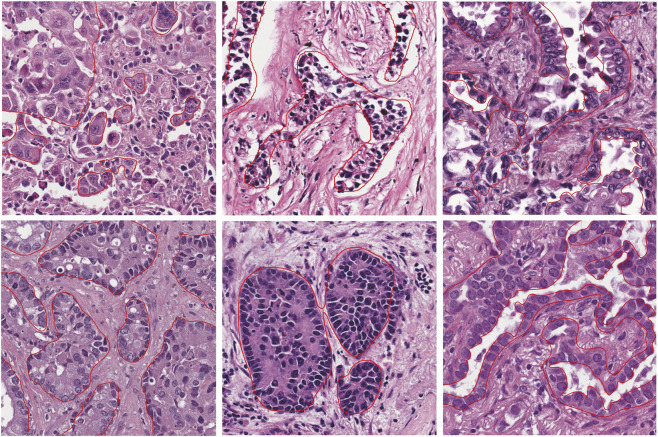
Visualization of tumor parenchyma segmentation model performance across various histological subtypes including solid, papillary, and acinar patterns. The tumor parenchyma regions automatically segmented by the U^2^-NetP model are outlined with red bounding boxes.

The inherent morphological complexity of LUAD presents substantial challenges for segmentation. LUAD encompasses multiple histological subtypes, including lepidic, acinar, papillary, micropapillary, and solid patterns, which demonstrate significant differences in tumor cell arrangement, tissue architecture, and stroma-to-tumor ratio. To visually assess the U^2^-NetP model’s performance under these complex pathological conditions. Figure 7 presents its segmentation results across multiple representative scenarios.

The model achieved optimal performance in solid and papillary patterns, where tumor cells form densely connected regions with relatively clear boundaries, resulting in accurate and continuous contours. However, performance degradation was observed in lepidic growth areas, regions with hyperchromatic tumor nuclei, and diffusely infiltrative zones. These challenging regions are characterized by tight tumor-stroma interweaving, low tissue contrast, and poorly defined boundaries, leading to occasional missed detection of scattered tumor foci. This represents a current limitation of the model.

Despite these challenges, U^2^-NetP maintained robust segmentation performance across most cases, substantially outperforming other compared models. This effectiveness stems from its nested U-shaped architecture and residual U-block design, which enable multi-level feature extraction to address heterogeneity and ambiguous boundaries.

### Model validation and efficiency analysis for lymphocyte recognition

Lymphocyte detection within the segmented stromal regions represents one of the most challenging tasks in the entire analytical pipeline. This task encounters multiple difficulties including substantial morphological variability among cells, dense cellular distributions, frequent confusion with imaging artifacts, and close visual resemblance to tumor cells. The implemented YOLOv7-based detection framework addresses these challenges through multi-scale prediction and feature pyramid networks, substantially improving small-target detection sensitivity. On the test set, the final model achieved a precision of 77.89%, recall of 79.81%, F1-score of 78.84%,and mAP@0.5 81.16%. Notably, the mAP@0.5 performance demonstrates a high level of accuracy and is particularly outstanding on our custom dataset. In regions with high-density lymphocyte aggregation specifically, the optimized non-maximum suppression strategy effectively reduced both duplicate and missed detections.

The YOLO architecture is widely acknowledged for its exceptional processing speed in real-time object detection applications. Its core advantage stems from formulating object detection as a single regression problem, which enables direct prediction of target locations and categories through a single forward propagation pass. This design eliminates the computationally expensive region proposal stages characteristic of traditional two-stage detectors. Through successive version iterations, the YOLOv7 model employed in this work preserves the essential design principles of the YOLO architecture while achieving enhanced computational efficiency. Experimental results demonstrate that the model maintains high processing throughput when analyzing WSIs of varying dimensions (Table 3).

**TABLE 3:** Efficiency analysis of YOLOv7 model for processing WSIs of different sizes.

WSIs size (MB)	Sample size (N = 98)	Average total processing time (Min)	Average core inference time (min)	Inference time percentage (%)
<100	20	2.38 ± 0.57	2.15 ± 0.65	90.26
100–300	23	4.21 ± 0.98	3.81 ± 0.87	90.03
300–600	25	8.66 ± 1.25	7.81 ± 1.67	90.08
600–1000	18	17.51 ± 3.51	16.06 ± 3.23	91.73
>1000	12	26.50 ± 4.36	24.55 ± 2.90	92.68

**FIGURE 8:**
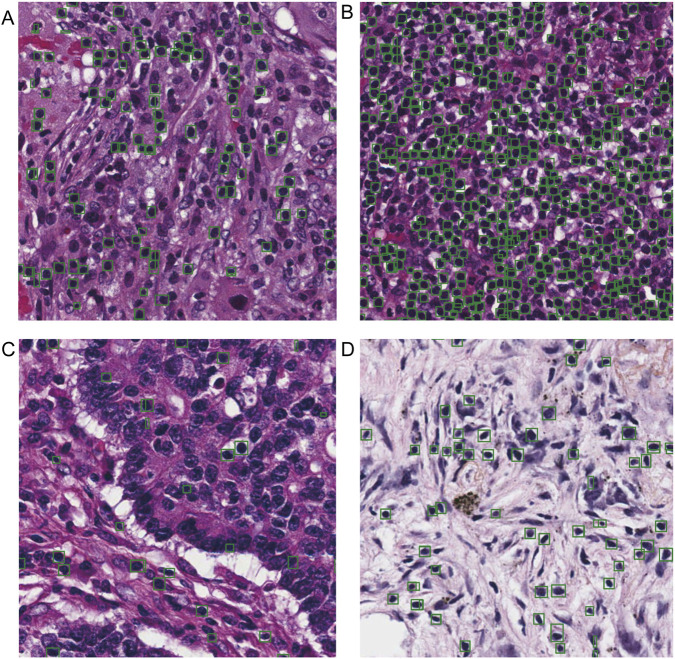
Segmentation performance of the lymphocyte detection model across different challenging scenarios. **(A)** Typical clear regions. **(B)** High-density clustered regions. **(C)** Regions with cellular morphological confusion. **(D)** Challenging areas with faint staining. Green boxes indicate the lymphocyte detection results from the automated pipeline.

Building on its architectural advantages, the YOLOv7 model exhibits strong applicability across diverse pathological specimens. In typical regions with clear TILs distribution and moderate cell density, the model achieves accurate identification and segmentation (Figure 8A). In high-density TILs regions, particularly within tertiary lymphoid structures, it maintains high recall despite challenges posed by cell overlapping and boundary adhesion, demonstrating robust analytical capability in cell aggregation areas (Figure 8B).

In peritumoral regions where TILs and tumor cells exhibit high morphological similarity, feature space aliasing results in blurred decision boundaries. Under these conditions, the confidence-based decision mechanism is often triggered, suppressing prediction boxes with confidence scores below the preset threshold and consequently producing localized missed detections (Figure 8C).

To assess model performance under common clinical challenges including staining inconsistency and section fading, evaluation was conducted on slides with similar characteristics. As shown in Figure 8D, the model maintains stable performance under these conditions, accurately identifying, localizing, and segmenting TILs even in faintly stained sections, confirming its generalization capability and robustness when presented with challenging pathological cases.

### Validation of agreement between automated and manual TILs assessment

**FIGURE 9:**
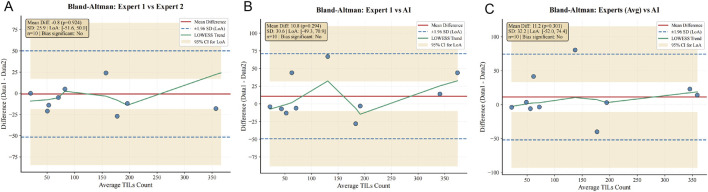
Bland-Altman plots for TILs count agreement. **(A)** Pathologist 1 vs. Pathologist 2. **(B)** Pipeline vs. Pathologist 1. **(C)** Pipeline vs. Pathologists’ average. Solid line: mean bias; dashed lines: 95% limits of agreement.

The ICC analysis revealed excellent agreement between the two pathologists in TILs count estimation (ICC = 0.981, 95% CI: 0.930–0.995). The pipeline also demonstrated high agreement with each pathologist individually (ICC = 0.970 with Expert 1, 95% CI: 0.910–0.990; ICC = 0.965 with Expert 2, 95% CI: 0.900–0.985), with an overall ICC of 0.972 (95% CI: 0.920–0.990) (Table 4). Bland-Altman plots further visualized these comparisons, showing minimal bias between experts and acceptable, albeit slightly systematic, bias between the pipeline and human raters (Figure 9).

**TABLE 4:** Agreement analysis for TILs counts among raters.

Comparison group	ICC value	95% confidence interval	Agreement strength
Pathologist 1 vs. Pathologist 2	0.981	0.930–0.995	Excellent
AI pipeline vs. Pathologist 1	0.970	0.910–0.990	Excellent
AI pipeline vs. Pathologist 2	0.965	0.900–0.985	Excellent
Overall	0.972	0.920–0.990	Excellent

ICC, intraclass correlation coefficient (two-way random-effects model for absolute agreement). Agreement strength was interpreted per Cicchetti (1994): <0.40 poor; 0.40–0.59 fair; 0.60–0.74 good; ≥0.75 excellent.

For categorical grading, Cohen’s kappa indicated perfect agreement between the two pathologists (κ = 1.000, 95% CI: 1.000–1.000). The agreement between the pipeline and the pathologists was substantial (κ ≈ 0.833, 95% CI: 0.500–1.000), with an overall Fleiss’ kappa of 0.889 (95% CI: 0.667–1.000) (Table 5).

**TABLE 5:** Agreement analysis for TILs semi-quantitative grading.

Comparison group	Kappa value (κ)	95% confidence interval	Agreement strength
Pathologist 1 vs. pathologist 2	1.000	1.000–1.000	Almost perfect
AI pipeline vs. pathologist 1	0.833	0.500–1.000	Almost perfect
AI pipeline vs. pathologist 2	0.833	0.500–1.000	Almost perfect
Overall (Fleiss’Kappa)	0.889	0.667–1.000	Almost perfect

Agreement strength was interpreted per Landis and Koch (1977): <0.00 poor; 0.00–0.20 slight; 0.21–0.40 fair; 0.41–0.60 moderate; 0.61–0.80 substantial; 0.81–1.00 almost perfect. Grading was based on three categories: low (<100 cells/HPF), moderate (100–300 cells/HPF), high (>300 cells/HPF).

### External validation on an independent cohort

To assess the generalizability of our pipeline, we evaluated it on an independent external cohort. For tumor parenchyma segmentation, the model demonstrated robust and consistent performance across all five histological subtypes (Table 6), with Dice coefficients ranging from 0.8509 to 0.9178.

**TABLE 6:** Performance of tumor parenchyma segmentation on external validation WSIs.

Growth pattern	No. of patches	Dice coefficient (mean ± SD)	Precision (mean ± SD)	Recall (mean ± SD)
Solid	169	0.8571 ± 0.2664	0.9207 ± 0.2550	0.8896 ± 0.1536
Papillary	36	0.9178 ± 0.0396	0.9631 ± 0.0168	0.8802 ± 0.0651
Complex acinar	42	0.9008 ± 0.1480	0.9485 ± 0.1514	0.8860 ± 0.0715
Acinar	36	0.8909 ± 0.0898	0.9454 ± 0.0239	0.8186 ± 0.1356
Lepidic	36	0.8509 ± 0.0629	0.9272 ± 0.0400	0.7661 ± 0.1027

**FIGURE 10:**
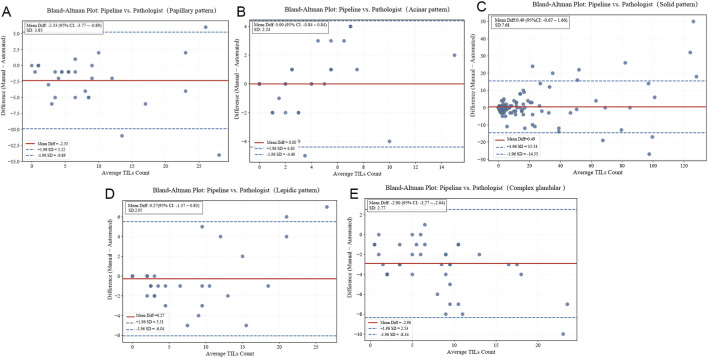
Bland‑Altman plots for TILs counts across five histological subtypes. **(A)** Papillary, **(B)** Acinar, **(C)** Solid, **(D)** Lepidic, **(E)** Complex glandular. Solid red line: mean bias; dashed blue lines: 95% limits of agreement (mean ± 1.96 SD).

For TILs detection, the agreement analysis between automated and manual counting on the external validation cohort demonstrated good-to-excellent concordance across all histological subtypes. As detailed in Table 7, the ICC values ranged from 0.796 (complex acinar) to 0.920 (lepidic). Notably, the model maintained excellent agreement (ICC >0.91) in challenging solid and lepidic growth patterns (Figure 10). These results confirm that the pipeline’s TILs quantification remains highly consistent with expert assessment on independent, externally sourced data.

**TABLE 7:** External validation of TILs detection agreement.

Growth pattern	Number of patches evaluated	ICC (95% confidence interval)	Agreement strength
Solid	169	0.918 (0.870–0.953)	Excellent
Papillary	36	0.846 (0.715–0.925)	Good to excellent
Complex acinar	42	0.796 (0.721–0.879)	Excellent
Acinar	36	0.804 (0.653–0.908)	Good to excellent
Lepidic	36	0.920 (0.866–0.947)	Excellent

## Discussion

The biological significance and clinical implications of TILs in the immune microenvironment of LUAD have remained a focal point of research interest. The absolute count of TILs serves as a critical metric for assessing the extent of immune infiltration, with higher densities correlating significantly with prolonged overall survival and improved responses to immunotherapy, reflecting the host’s fundamental antitumor immune capacity (Han et al., 2024). The spatial architecture of TILs also holds pivotal importance, particularly the formation of organized clusters such as tertiary lymphoid structures. These organized formations represent a morphological manifestation of a functional immune microenvironment and are closely associated with enhanced antitumor activity and favorable clinical outcomes (Lopez de Rodas et al., 2022). Computational pathology integrates artificial intelligence with histopathological image analysis, offering a novel approach to deeply mine potential immune-related features from pathological images (Madabhushi and Lee, 2016). This enables the characterization of tumor immune microenvironment status, prediction of immunotherapy response, and assessment of patient prognosis. Supervised learning based on high-quality annotated data has become the predominant paradigm for achieving high-precision modeling in this field (Del Amor et al., 2023). However, the acquisition of large-scale annotated datasets remains a critical bottleneck hindering research progress. In this context, the automated pipeline developed in this study provides researchers with an efficient and lightweight solution. Its compact model design substantially reduces computational resource requirements and provides a high-quality automated tool to facilitate the achievement of the above research objectives.

This study developed a fully automated deep learning pipeline to address the critical need for high-quality annotated data in TILs research for LUAD (Bocchialini et al., 2022; Jung et al., 2022). Following a modular design philosophy, the pipeline processes WSIs through three logically sequential stages. The workflow begins with tissue contour detection to establish spatial reference frameworks, progresses to tumor parenchyma segmentation for defining histological boundaries, and culminates in precise TILs identification within the delineated stromal regions. This strategic pairing of specialized computational architectures with distinct analytical tasks enables seamless integration across scales, from tissue-level mapping to cellular-level detection. The implemented system efficiently captures comprehensive TILs spatial distributions while generating high-quality annotations that establish a robust foundation for subsequent computational pathology research.

The performance variation across the three core modules reflects the distinct technical challenges inherent to their respective biological complexities. For tissue contour segmentation, the high contrast between target and background regions facilitates expert-level accuracy using an optimized OpenCV-based image processing pipeline. In contrast, tumor parenchyma segmentation and TILs identification encounter more substantial obstacles due to profound tumor heterogeneity. LUAD comprises multiple histological subtypes, such as lepidic, acinar, papillary, micropapillary, and solid patterns (Travis et al., 2011), which exhibit significant morphological diversity. This inter-subtype heterogeneity, combined with boundary ambiguity resulting from invasive growth patterns, presents fundamental challenges to accurate parenchyma-stroma separation.

To address this complex task, this study employed the U^2^-NetP architecture, whose performance stems from its unique approach to handling scale variation in medical image segmentation (Xuebin et al., 2020; Azad et al., 2024). The synergistic design of its nested U-shaped structure and residual U-blocks (RSUs) enables genuine multi-scale feature extraction and fusion. In particular, deep RSU modules utilize dilated convolutions to enlarge the receptive field, capturing global morphological features such as collagen fiber orientation and necrotic area distribution. In contrast, shallow RSU modules rely mainly on standard convolutions to extract local detail patterns including acinar structures and cell density distribution. This parallel multi-scale processing mechanism allows a single model to simultaneously interpret features spanning from microscopic acinar lumen formation to macroscopic acinar unit distribution patterns, thereby achieving precise identification of morphologically diverse parenchyma-stroma transition regions across LUAD subtypes.

During model training, input images were uniformly processed at a resolution equivalent to ×5objective magnification. Although this scale sacrifices certain cellular-level textural details, it retains sufficient tissue-level architectural information to enable accurate tumor parenchyma segmentation. This resolution strategy is grounded in the established principle that structural organization at the tissue level often provides greater discriminative power for parenchyma-stroma segmentation than cellular texture features alone (Zunair and Ben Hamza, 2021; Yang et al., 2025). Guided by this principle, the implemented low-resolution approach strategically balances precision with computational efficiency. This design not only reduces processing requirements but also accommodates the varying information density needs of different analytical tasks. In practice, the method demonstrated consistent segmentation performance and reliably identified invasive foci even in cases with poorly defined boundaries.

The particularly challenging task of TILs detection involves difficulties arising from small cell size, dense cellular distribution, and morphological similarity to tumor cell nuclei (Abhishek et al., 2023; Wu et al., 2023). The YOLOv7 architecture (Chien-Yao Wang and Liao, 2023) was selected as the optimal solution in this study, achieving an effective balance between detection speed and accuracy through its efficient multi-scale feature fusion and single-stage detection paradigm. Model effectiveness originates from its capacity to simultaneously capture local cellular textures and global distribution patterns. This capability is realized through the synergistic operation of its Feature Pyramid Network (FPN) (Lin et al., 2017) and Path Aggregation Network (PAN) (Shu Liu et al., 2018) which effectively integrate shallow fine-grained features with deep semantic information. Additionally, the decoupled detection head independently optimizes classification and regression tasks, enabling precise bounding box localization of lymphocytes (Cao et al., 2023).

While two-stage detectors such as Mask R-CNN may achieve superior accuracy on certain public datasets, their substantial computational demands and corresponding limitations in inference speed hinder application to large-scale WSIs analysis in clinical environments (Felfeliyan et al., 2022; Kanadath et al., 2025). Experimental results indicate that model selection in medical image analysis should be guided by comprehensive validation against specific task requirements, rather than by the conventional assumption that two-stage detectors inherently provide superior performance. By maintaining competitive detection accuracy while delivering significantly accelerated inference, YOLOv7 constitutes the optimal architecture for TILs detection in this study (Demetriou et al., 2023; Son and Ahn, 2025).

The modular pipeline design implemented in this study demonstrates that decomposing complex pathological image analysis into specialized subtasks, with each addressed by its most suitable architecture, produces superior overall performance compared to employing a single universal model (Qin et al., 2025; Moor et al., 2023). This approach enables the selection of precisely tailored technical solutions for the distinct challenges presented at each analytical stage. Specifically, for tissue contour detection as a foundational step, algorithmic stability and computational efficiency were prioritized, leading to the adoption of a lightweight yet robust image processing pipeline. For tumor parenchyma segmentation, which must accommodate complex morphological variations and ambiguous boundaries, U^2^-NetP was selected for its exceptional multi-level feature fusion capabilities. For TILs detection as a small-target recognition task, YOLOv7 provided the optimal balance between precision and inference speed.

This task-decomposition strategy enhances both model development efficiency and training effectiveness while ensuring accuracy and stability throughout the analytical workflow through systematically addressing each hierarchical level with targeted solutions. The approach fully leverages the distinct architectural strengths of different models within their specific contexts, thereby ensuring the reliability and robustness of the final integrated output. Furthermore, this modular framework aligns with established engineering principles for complex systems, facilitating future functional expansion and maintenance (Prorok, 2025).

The pipeline’s lightweight architecture enables efficient operation on standard GPU hardware, significantly improving accessibility. It offers researchers in related fields a practical tool for digital pathology studies in lung adenocarcinoma, while actively advancing the integration and application of artificial intelligence technologies within conventional pathological practice.

To address the need for benchmarking against biological reality, we rigorously compared our automated outputs with independent semi-quantitative assessments from two pathologists. The excellent inter-observer agreement first established a highly consistent human reference standard (Oisakede et al., 2025). Critically, our pipeline showed equally high agreement with each expert individually and substantial agreement in categorical grading, demonstrating that its quantitative outputs closely align with expert pathological judgment. Bland-Altman plots confirmed this strong agreement while revealing a modest, systematic underestimation by the model, likely reflecting its calibrated sensitivity to minimize false positives in complex regions. These results collectively affirm that the TILs maps generated by our pipeline are biologically plausible, as they reliably reproduce the assessments of experienced pathologists. Therefore, this automated tool provides a robust and scalable foundation for subsequent studies aiming to uncover clinically relevant spatial patterns of TILs in large cohorts (Ding et al., 2022).

## Limitations and future directions

This study has several limitations, beginning with the methodology for external validation. The annotations in the independent hospital cohort consisted of point-like circles marking lymphocyte centers, whereas our model outputs bounding boxes. This discrepancy in annotation granularity precluded a rigorous, object-level evaluation using metrics such as mAP@0.5, which requires precise spatial overlap. To address this, we adopted an alternative patch-based counting agreement analysis, comparing the total TILs counts within corresponding image patches between the model and pathologists. While this approach effectively validates the model’s quantitative accuracy in estimating lymphocyte counts, which is a clinically paramount metric, it does not fully assess its precision in single-cell localization. Future studies would benefit from pixel-level annotations on external sets to enable comprehensive spatial evaluation. This limitation also underscores a broader challenge in computational pathology: the lack of standardized annotation protocols across institutions, which is crucial for the equitable benchmarking and clinical translation of AI models (Montezuma et al., 2023; Xu et al., 2024).

Beyond this, this study has further limitations. First, the scale of the training dataset remains constrained, with particularly insufficient annotated cases representing the full spectrum of LUAD histological subtypes, which may impact model generalizability across diverse pathological manifestations. While the addition of independent internal and external validation cohorts strengthens the evidence for generalizability, the current scale of these cohorts limits the granularity of performance characterization, particularly regarding the distribution of metrics across individual slides. Future work should prioritize the assembly of larger, multi-institutional validation cohorts. Second, the automated pipeline is primarily focused on morphological annotation, and the generated data have not yet been systematically linked to downstream biological insights (e.g., specific gene expression or pathway activity (Ma et al., 2025)) or clinical endpoints (e.g., immune subtyping (Xie et al., 2025), tumor grade, or overall survival (Han et al., 2024)). This restricts the translational potential of the current findings. Additionally, technical challenges remain in model performance. Although the U^2^-NetP segmentation model demonstrates robust overall performance, it exhibits limitations in precisely delineating boundaries in certain complex subtypes characterized by ambiguous tissue interfaces. Furthermore, model effectiveness remains substantially dependent on extensive manual annotation. For TILs detection, the YOLOv7-based approach, while efficient, continues to face challenges in high-density cellular regions, where both missed detections and false positives occur.

To address these limitations, several promising research directions are proposed. We plan to conduct TILs-focused clinical studies using this annotation model to further evaluate its real-world translational utility. Architecturally, integrating emerging frameworks such as Vision Transformers (Vaswani et al., 2017) could enhance global context modeling, while incorporating boundary-optimized loss functions may improve segmentation precision at challenging tissue interfaces. From a methodological perspective, self-supervised (Wang et al., 2021) and semi-supervised learning strategies (Qi et al., 2024) offer considerable potential. By leveraging large-scale unannotated WSIs for pretraining, models can learn fundamental histomorphological representations, subsequently requiring only limited fine-annotated data for task-specific adaptation. This paradigm shift could substantially reduce the annotation burden in future model development, thereby improving both the efficiency and accessibility of automated annotation pipelines.

## Conclusion

This study presents an integrated and lightweight deep learning pipeline for the automated annotation of tissue contours, tumor parenchyma, and TILs in H&E-stained WSIs of LUAD. Experimental validation, including rigorous internal testing and independent external validation, demonstrates robust and generalizable performance across the primary annotation tasks. Critically, the automated TILs quantifications showed excellent agreement with independent pathologist assessments, confirming the biological plausibility of the generated annotations. The pipeline provides an efficient and reliable solution for large-scale pathological data processing, establishing a high-quality annotated data foundation for computational pathology research. Its practical utility is further enhanced by a modular and lightweight architecture, which lowers computational barriers and facilitates accessibility for both research and future translational applications.

## Data Availability

The source code implementing the proposed deep learning framework is publicly available at: https://github.com/YHRan/TILs.git.
