# Correction: An integrated automated deep learning framework for annotating tumor-infiltrating lymphocytes in lung adenocarcinoma pathology

**DOI:** 10.3389/fbinf.2026.1841924

**Published:** 2026-04-30

**Authors:** Xia Li, Kang-Lai Wei, Zhao-Quan Huang, Zi-Yan Huang

**Affiliations:** 1 Department of Pathology, The First Affiliated Hospital of Guangxi Medical University, Nanning, Guangxi, China; 2 Department of Pathology, The Second Affiliated Hospital of Guangxi Medical University, Nanning, Guangxi, China; 3 Health Management Department, The First Affiliated Hospital of Guangxi Medical University, Nanning, Guangxi, China

**Keywords:** automated annotation, deep learning, lung adenocarcinoma, pathology, tumor-infiltrating lymphocytes

In the published article, there was an error in Table 5 as published. The table should have presented the agreement analysis for TILs semi-quantitative grading based on Kappa**.** The corrected Table 5 and its caption appear below.

**TABLE 5:** Agreement analysis for TILs semi-quantitative grading.

Comparison group	Kappa value (κ)	95% confidence interval	Agreement strength
Pathologist 1 vs. pathologist 2	1.000	1.000–1.000	Almost perfect
AI pipeline vs. pathologist 1	0.833	0.500–1.000	Almost perfect
AI pipeline vs. pathologist 2	0.833	0.500–1.000	Almost perfect
Overall (Fleiss’Kappa)	0.889	0.667–1.000	Almost perfect

Agreement strength was interpreted per Landis and Koch (1977): <0.00 poor; 0.00–0.20 slight; 0.21–0.40 fair; 0.41–0.60 moderate; 0.61–0.80 substantial; 0.81–1.00 almost perfect. Grading was based on three categories: low (<100 cells/HPF), moderate (100–300 cells/HPF), high (>300 cells/HPF).

The original article has been updated.

