# Correction: An integrated automated deep learning framework for annotating tumor-infiltrating lymphocytes in lung adenocarcinoma pathology

**DOI:** 10.3389/fbinf.2026.1920827

**Published:** 2026-07-01

**Authors:** Frontiers Production Office

**Affiliations:** Frontiers Media SA, Lausanne, Switzerland

**Keywords:** automated annotation, deep learning, lung adenocarcinoma, pathology, tumor-infiltrating lymphocytes

The Data Availability statement was erroneously given as “The raw data supporting the conclusions of this article will be made available by the authors, without undue reservation.” The correct Data Availability statement is “The source code implementing the proposed deep learning framework is publicly available at: https://github.com/YHRan/TILs.git”.

The original article has been updated.

