# Supplementary material for: An integrated automated deep learning framework for annotating tumor-infiltrating lymphocytes in lung adenocarcinoma pathology
Source: Front Bioinform. 2026 Mar 16;6:1764743. doi: 10.3389/fbinf.2026.1764743 (PMC13033600; doi:10.3389/fbinf.2026.1764743)
Supplement: Supplementary file 1 [file Supplementaryfile1.doc]

1. **Confusion Matrix**

To comprehensively evaluate model performance, this study introduces the concept of a confusion matrix. A confusion matrix is a 2×2 table that summarizes the predictions of a binary classification model. As shown in Figure 1, the matrix consists of four key components:


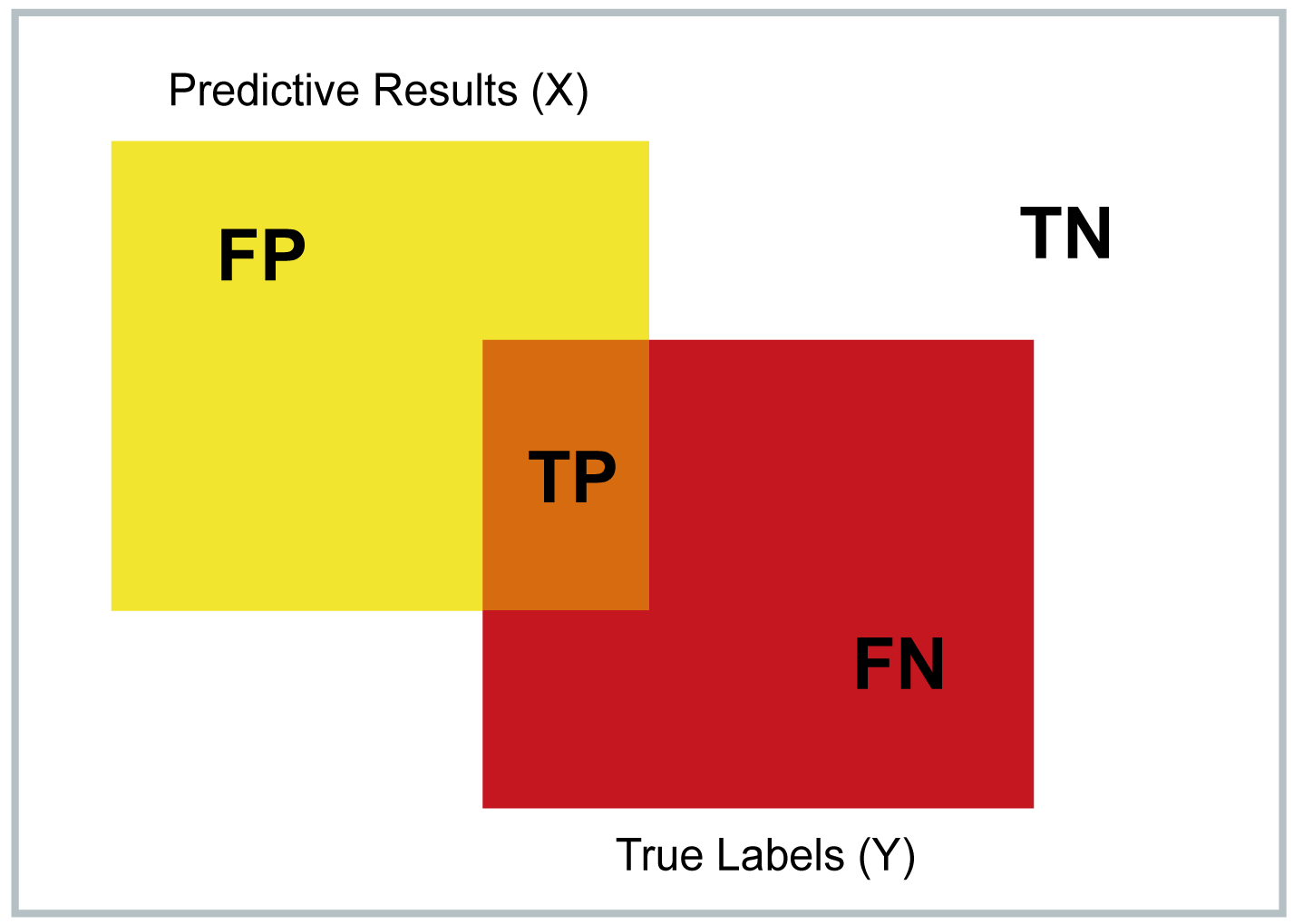


Figure 1 Diagram of the confusion matrix

True Positive (TP): The number of samples correctly predicted as positive by the model, i.e., cases where the model predicts positive and the actual label is also positive.

True Negative (TN): The number of samples correctly predicted as negative by the model, i.e., cases where the model predicts negative and the actual label is also negative.

False Positive (FP): The number of samples incorrectly predicted as positive by the model, i.e., cases where the model predicts positive but the actual label is negative.

False Negative (FN): The number of samples incorrectly predicted as negative by the model, i.e., cases where the model predicts negative but the actual label is positive.

1. **Key Metrics for Evaluating Deep Learning Model Performance**

Precision: Calculated as TP/(TP+FP), it represents the proportion of correctly predicted positive samples among all samples predicted as positive. Precision measures the accuracy of the model's positive predictions.

Recall: Calculated as TP/(TP+FN), it represents the proportion of correctly predicted positive samples among all actual positive samples. Recall measures the model's ability to identify positive instances.
